# Supplementary material for: EMP3 sustains oncogenic EGFR/CDK2 signaling by restricting receptor degradation in glioblastoma
Source: Acta Neuropathol Commun. 2023 Nov 7;11:177. doi: 10.1186/s40478-023-01673-z (PMC10629159; doi:10.1186/s40478-023-01673-z)
Supplement: Supplementary file 2 — Additional file 2. Supplementary figures. [file 40478_2023_1673_MOESM2_ESM.pdf]

Supplementary Figures

EMP3 sustains oncogenic EGFR/CDK2 signaling by restricting receptor degradation in glioblastoma

Acta Neuropathologica Communications

Corresponding author:
Stefan Pusch
Clinical Cooperation Unit Neuropathology
German Cancer Research Center
Im Neuenheimer Feld 280
69120 Heidelberg, Germany

E-mail: s.pusch@dkfz-heidelberg.de

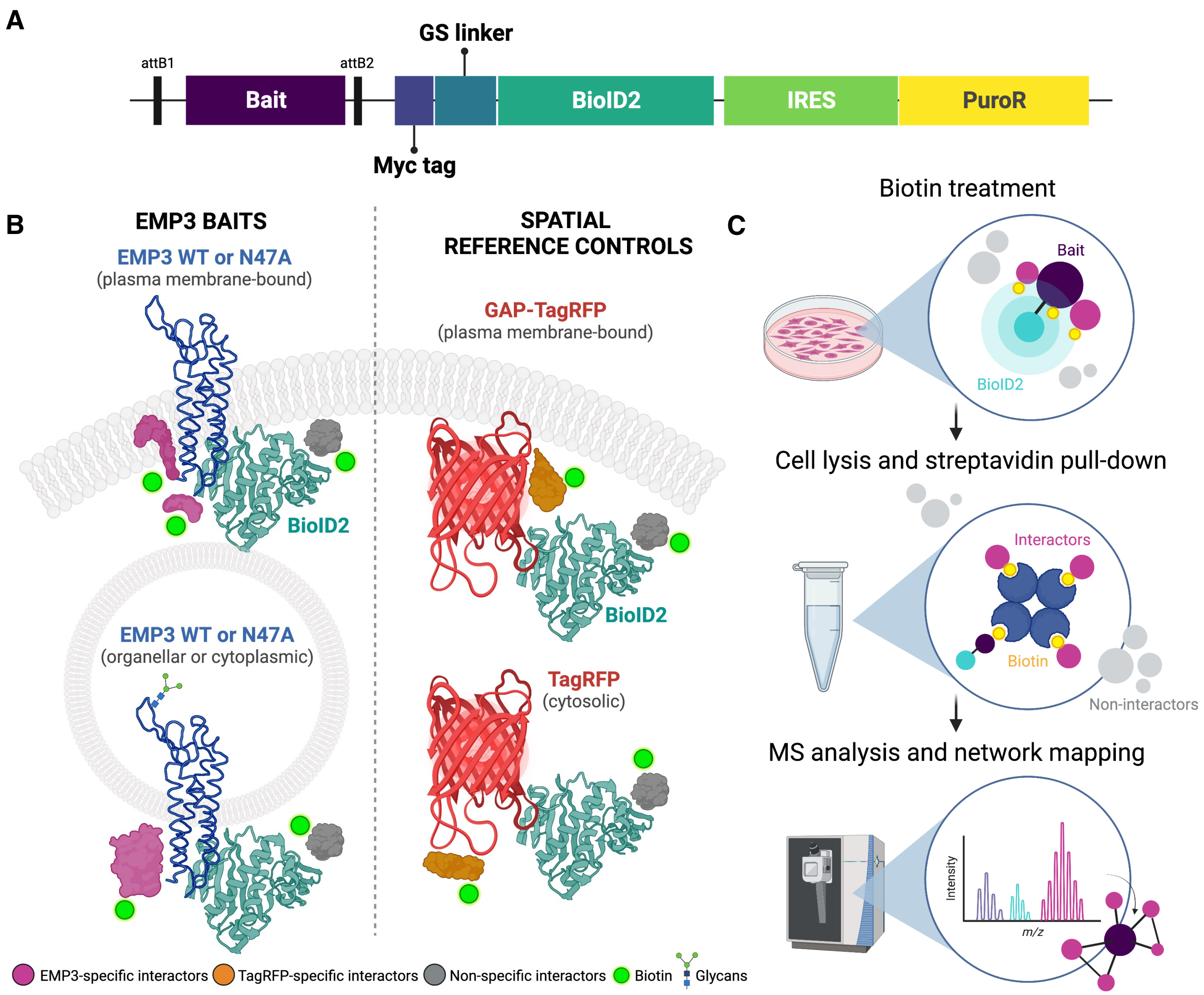

Figure S1. Experimental workflow and design of BioID2-based proximity labeling

A Schematic diagram of the Gateway construct expressing the bait protein fused to a C-terminal Myc glycine-serine (GS) linker-BioID2 tag. An IRES-PuroR cassette follows the coding sequence of the fusion protein. **B** Experimental design showing baits used in the BioID2 experiment. Wild-type and N47A mutant versions of EMP3 tagged with Myc-Linker-BioID2 at the C-terminal end were used as experimental baits. EMP3 baits may localize in the plasma membrane or within unidentified cytoplasmic compartments. TagRFP and GAP-TagRFP fusion proteins, which localize in cytoplasm and the plasma membrane, respectively, were used as spatial reference controls. **C** Workflow detailing experimental steps from biotin treatment to mass spectrometry (MS) analysis of identified EMP3-proximal proteins. U-118 cells stably transfected with BioID2 constructs were treated with 50 µM biotin for 18 hours to induce BioID2-mediated biotinylation. Biotinylated proteins were purified by streptavidin pull-downs, and the eluates were subjected to MS and network analysis.

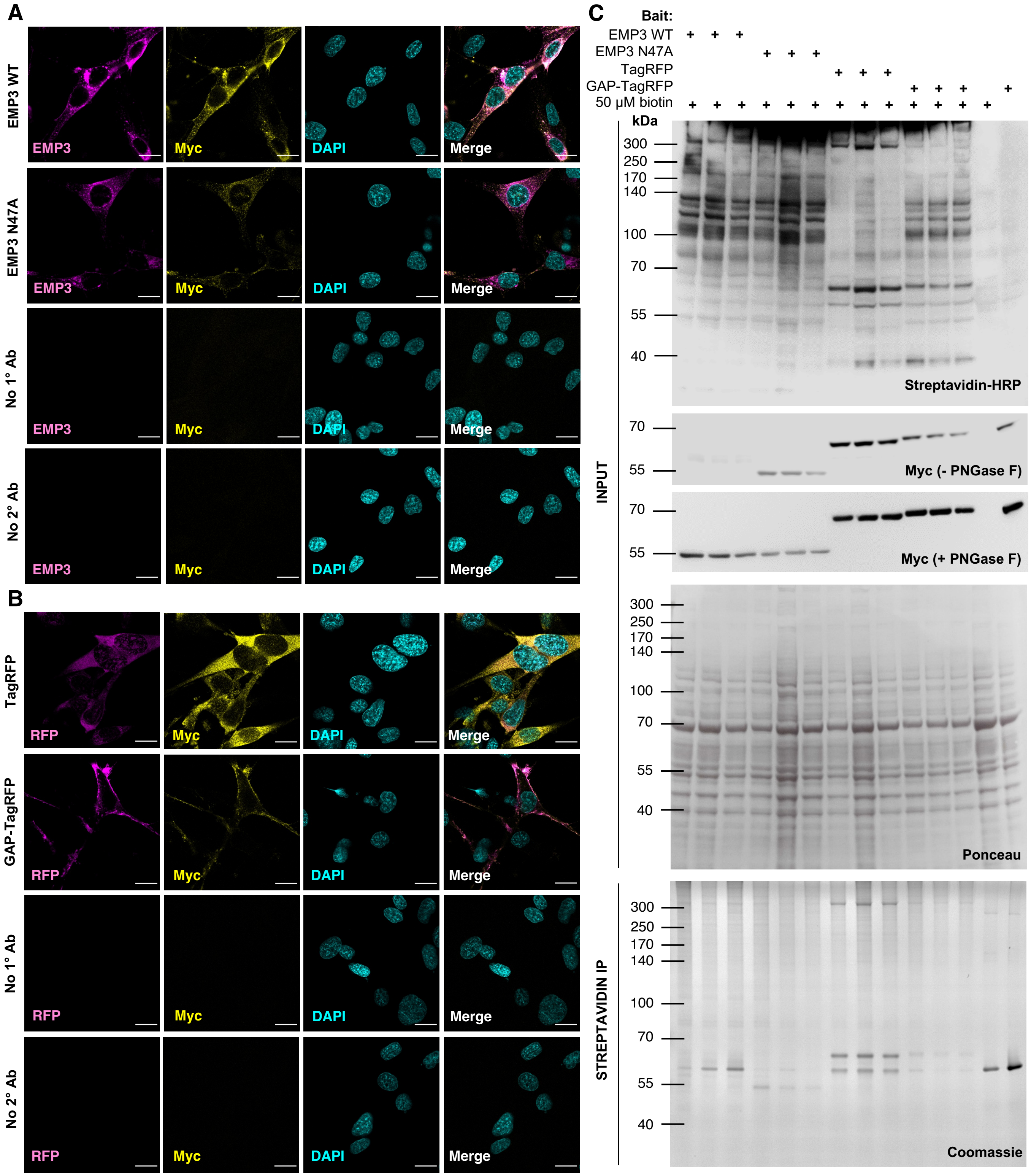

Figure S2. Validation of bait localization in U-118 cells stably transfected with BioID2 constructs
A Immunofluorescence (IF) staining of Myc-Linker-BioID2-tagged EMP3 WT and N47A proteins in U-118 cells. Antibodies against EMP3 (magenta) and Myc (yellow) were used to stain BioID2 fusion proteins. DAPI (cyan) was used for nuclear staining. Scale bar = 20 μm. **B** IF staining of Myc-Linker-BioID2-tagged TagRFP and GAP-TagRFP proteins in U-118 cells. RFP fluorescence (magenta) was visualized alongside Myc (yellow) and nuclear staining (DAPI). U-118 cells without either TagRFP constructs were used as controls. Scale bar = 20 μm. **C** Validation of BioID2-based proximity labeling. Bait expression was confirmed by immunoblotting for the Myc tag. Biotinylation of bait-proximal proteins was verified by streptavidin-HRP blots of the input lysate. Coomassie staining was performed on the eluates to confirm purification of biotinylated proteins.

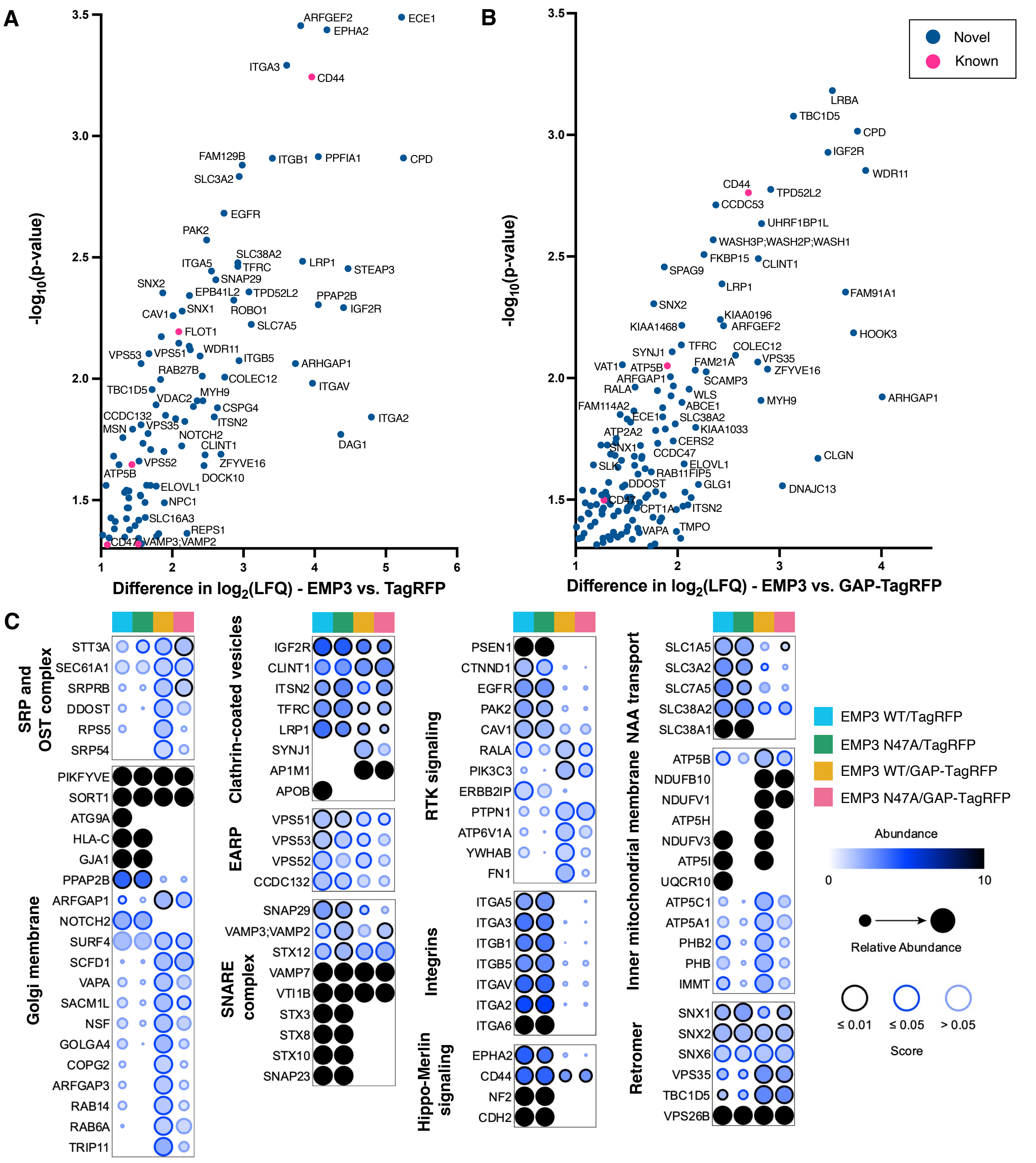

Figure S3. Enrichment of EMP3-proximal proteins in EMP3 WT/N47A baits versus spatial reference controls

A,B Scatter plots showing EMP3-proximal proteins (i.e. difference in log2(LFQ) ≥ 1, Welch's t-test p-value ≤ 0.05) identified using EMP3 WT as bait and TagRFP (A) or GAP-TagRFP (B) as the spatial reference control. Blue dots indicate novel EMP3-interacting partners, while pink dots represent previously identified EMP3 interactors based on the EMP3 yeast two-hybrid screen by Christians et al. and the BioGRID database (v4.4). **C** Dot plots showing relative enrichment of EMP3-proximal proteins, filtered and grouped in Cytoscape as previously described, across possible pairwise comparisons. Dot colors and sizes correspond to the difference in log2(LFQ) values of EMP3 baits versus spatial reference controls. The selected abundance cap automatically assigns a black fill color to proteins uniquely purified in EMP3 WT and N47A pull-downs while allowing visualization of the range of possible enrichment values. Border colors correspond to significance levels. All dot plots were generated using ProHits-viz.

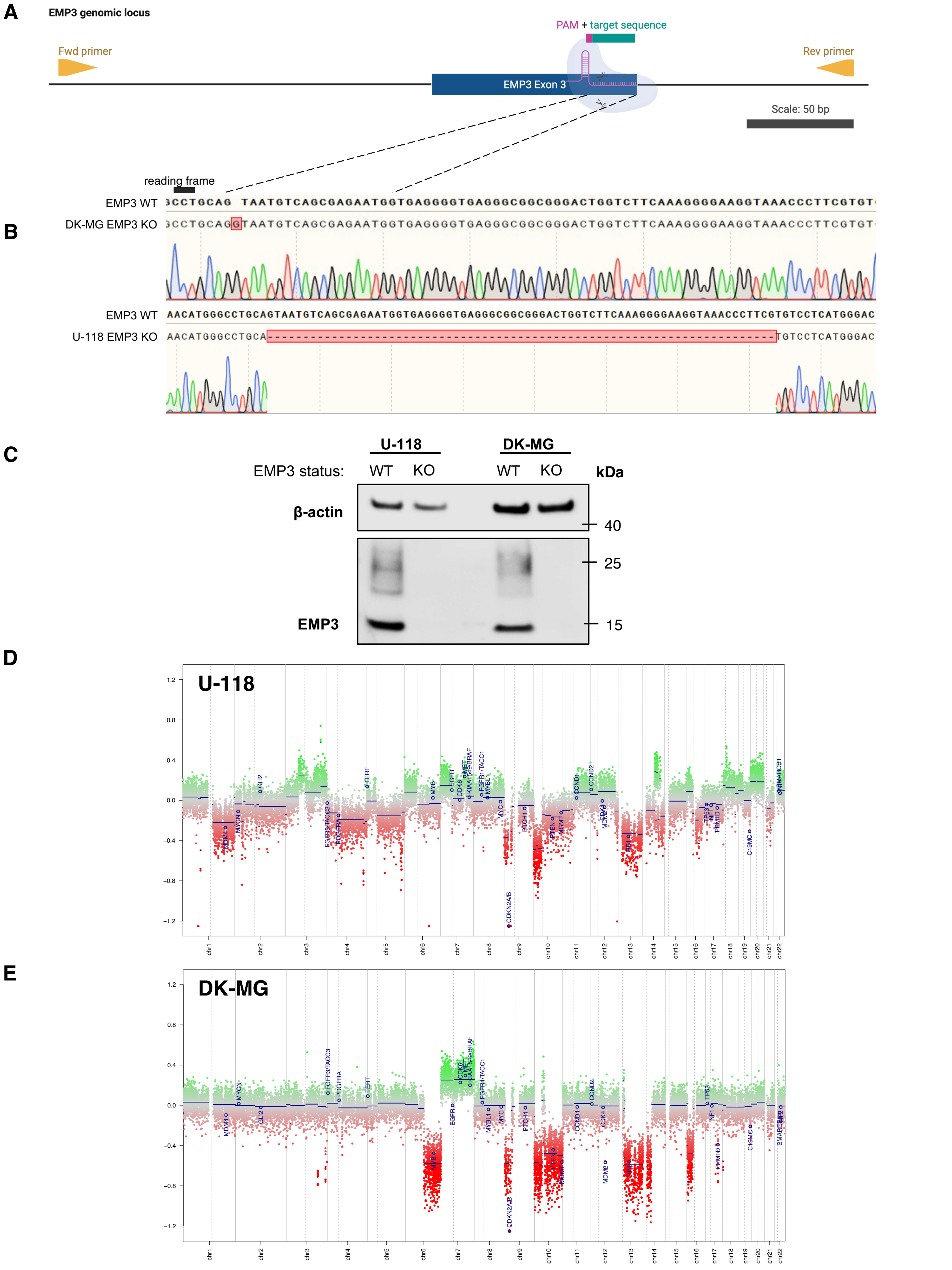

Figure S4. CRIPSR/Cas9-mediated EMP3 knockout and copy number profiling of U-118 and DK-MG cells
A Schematic diagram showing the EMP3 genomic locus containing Exon 3 flanked by intronic sequences on either side, and the relative positions of the guide RNA (gRNA) target sequence, PAM site, and primer-binding sites for forward (Fwd) and reverse (Rev) sequencing primers. Scale bar = 50 bp. **B** Sequencing results confirm the insertion of a single guanine nucleotide 2 bp downstream of the putative gRNA cut site in the DK-MG EMP3 KO cell line and a 71-bp deletion after the cut site in U-118 EMP3 KO cells. Both alterations should induce nonsense-mediated mRNA decay of the EMP3 transcript. **C** Western blots confirming proper EMP3 KO at the protein level in both cell lines. **D, E** Chromosomal copy number plots of U-118 (D) and DK-MG (E) cells.

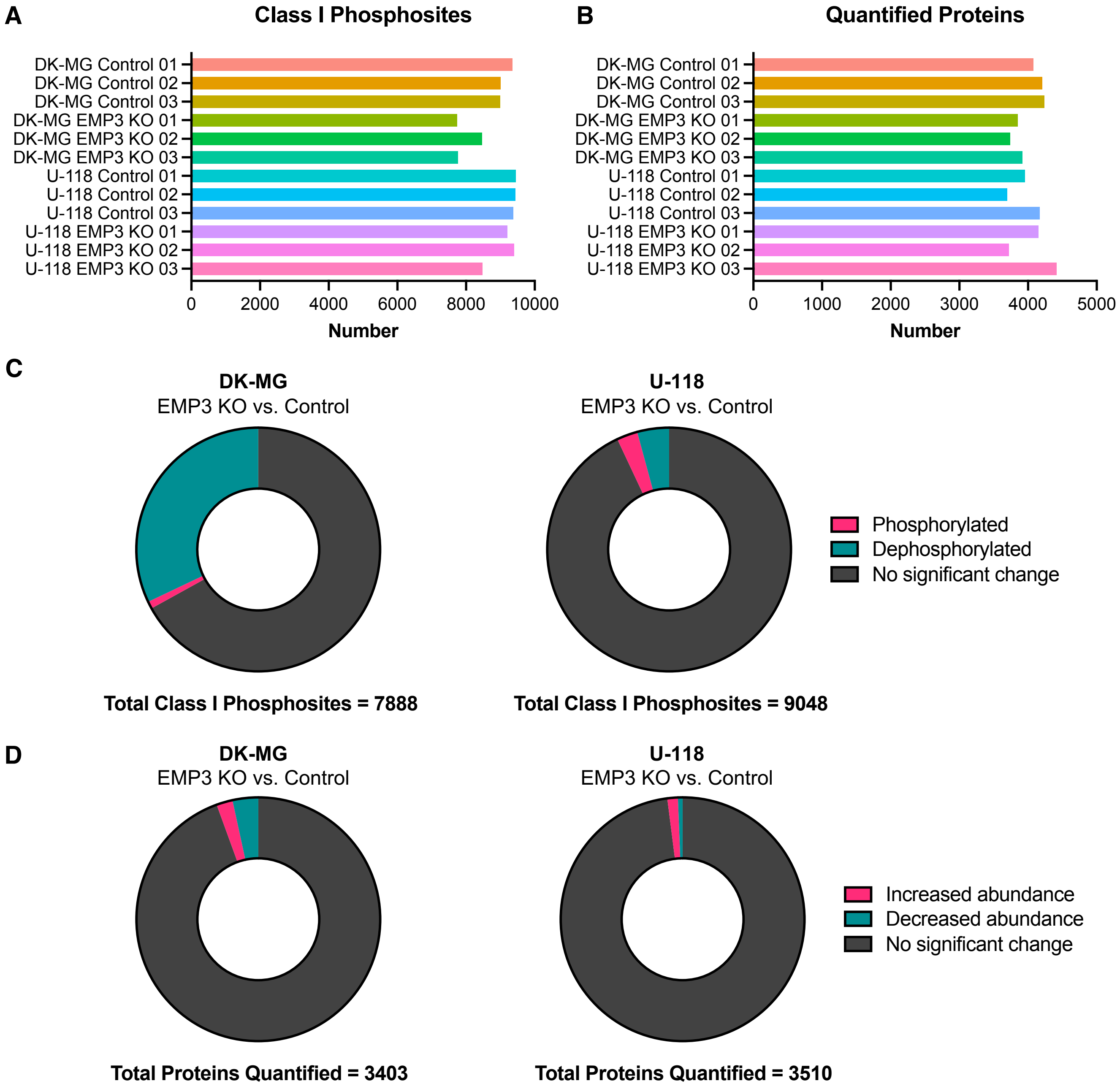

Figure S5. Number and type of phosphosites and proteins quantified by phosphoproteomics and full proteomics analysis

A,B Bar plots showing the total number of class I phosphosites (i.e., localization probability ≥ 0.75) identified (A) and total number of proteins quantified in each sample (B) after mass spectrometry analysis. **C** Donut charts showing the distribution of phosphorylation changes occurring in class I phosphosites that were detected in at least 2 out of 3 replicates of each condition (i.e., control or EMP3 KO) in DK-MG (left) and U-118 (right) cells. Phosphorylated - $\log_2$-FC ≥ 1, FDR p-value ≤ 0.05; Dephosphorylated - $\log_2$-FC ≤ -1, FDR p-value ≤ 0.05; No significant change – [$\log_2$-FC| < 1 and/or FDR p-value > 0.05. **D** Donut charts showing the distribution of proteins that were quantified in at least 2 out of 3 replicates of each condition (i.e., control or EMP3 KO) in DK-MG (left) and U-118 (right) cells. Increased abundance – $\log_2$-FC ≥ 1, FDR p-value ≤ 0.05; Decreased abundance – $\log_2$-FC ≤ -1, FDR p-value ≤ 0.05; No significant change – [$\log_2$-FC| < 1 and/or FDR p-value > 0.05.

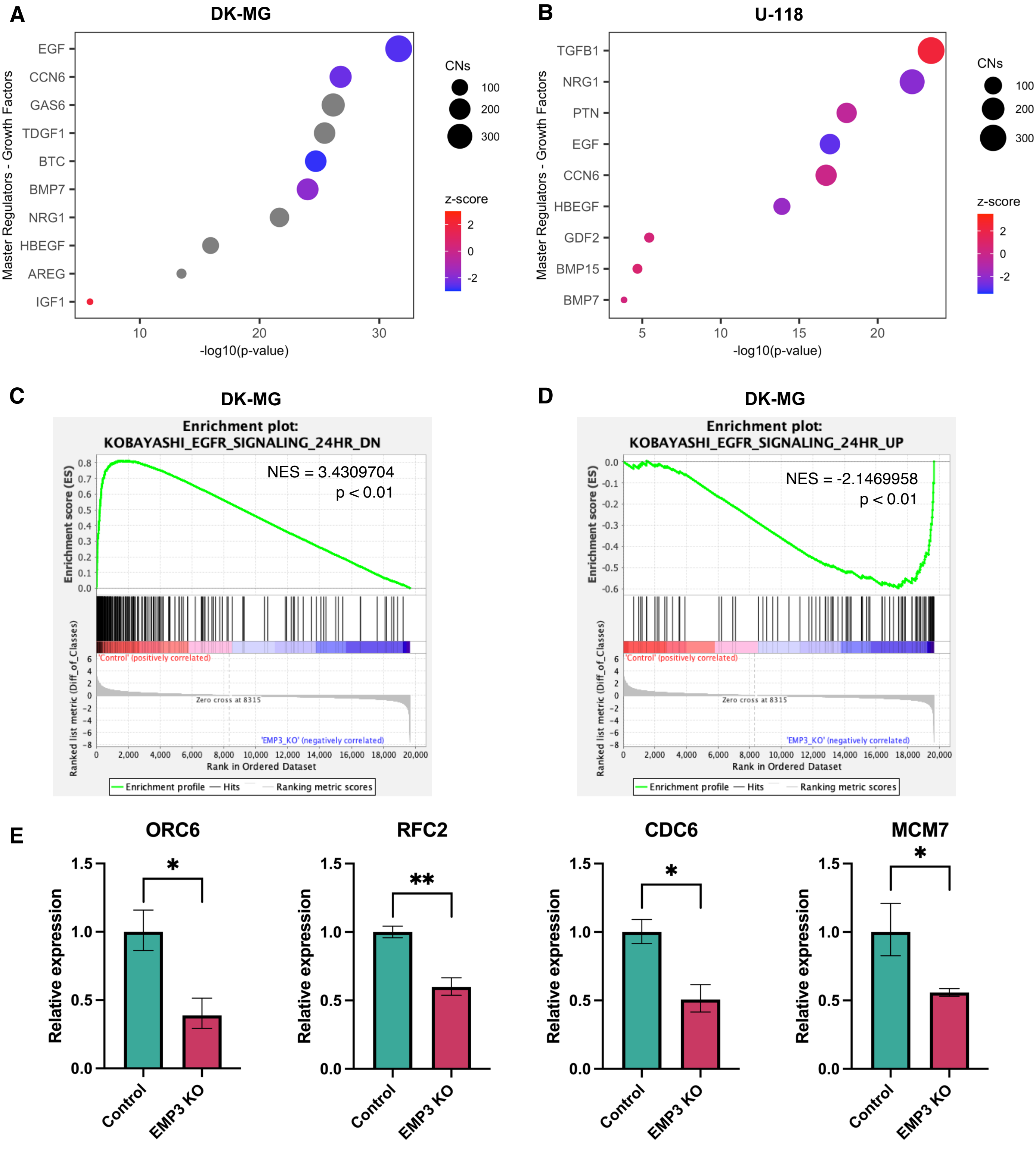

Figure S6. Inhibition of an EGF-dependent transcriptional program in DK-MG and U-118 EMP3 KOs

A, B Growth factors identified to be significantly enriched master regulators (MRs) based on DEGs in DK-MG (A) and U-118 (B) EMP3 KO relative to controls. MRs putatively regulating the input genes are listed on the y-axis and ordered according to significance. Circle sizes represent the number of associated causal networks (CNs) per MR, while the color scale indicates the activation z-score of each MR (red – active; blue– inactive). **C,D** GSEA analysis showing upregulation of the KOBAYASHI_EGFR_SIGNALING_24HR_DN (C) and KOBAYASHI_EGFR_SIGNALING_24HR_UP (D) gene sets in DK-MG control and EMP3 KO cells, respectively. Genes were sorted from left to right based on the difference of the $\log_2$ expression levels between control and EMP3 KO cells. Vertical black bars indicate the location of the genes contributing to the enrichment scores (ES). The ES, which indicate upregulation (ES > 0) or downregulation (ES < 0) of a certain gene, are plotted on the y-axis. NES: normalized enrichment score. **E**, qPCR results validating the downregulation of selected genes in DK-MG EMP3 KOs (unpaired one-tailed t-test; * P = < 0.05; ** P = < 0.01).

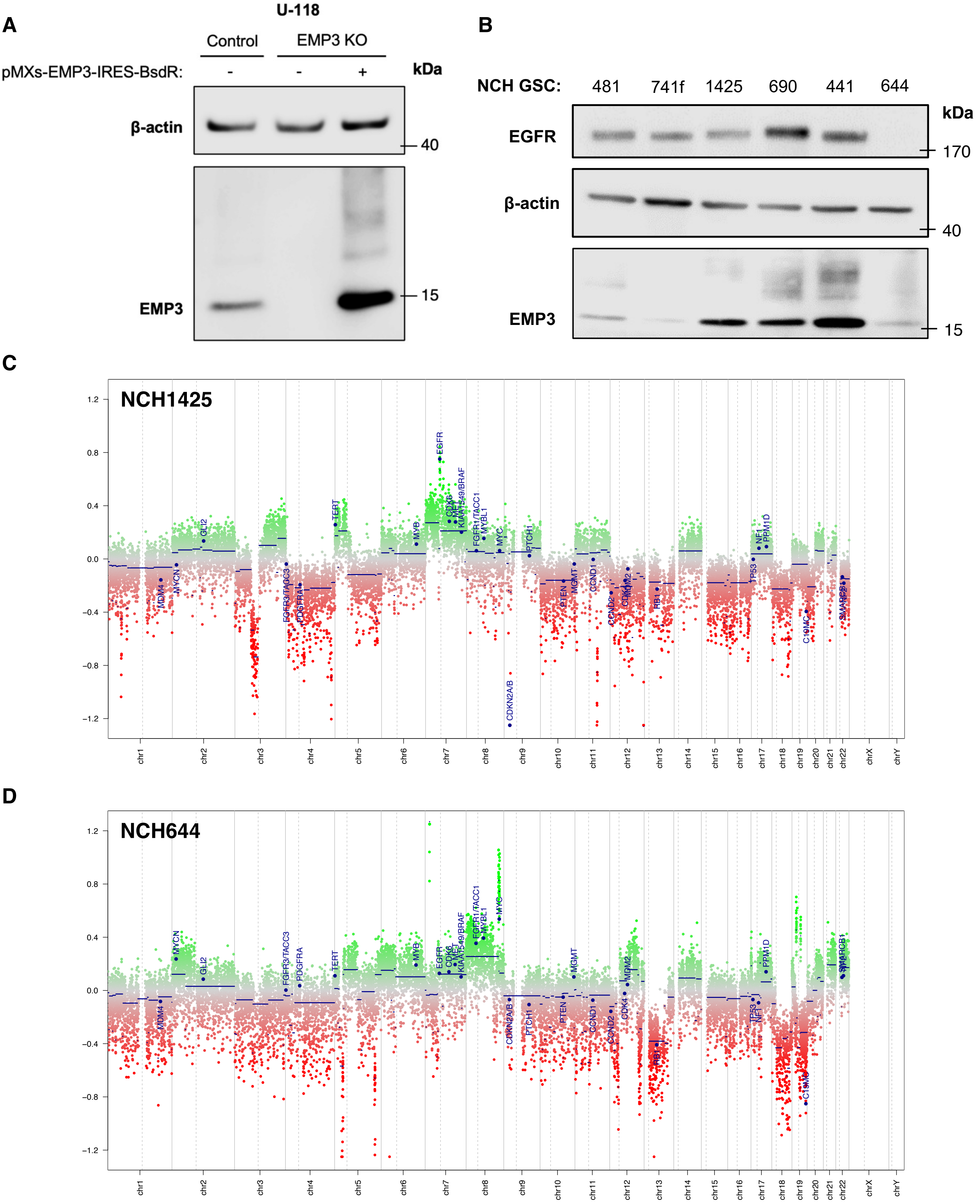

Figure S7. Validation and profiling of additional cellular models

A Western blots confirming proper re-expression of EMP3 in U-118 EMP3 KO cells. EMP3 KO cells were stably transfected with pMXs-EMP3-IRES-BsdR to restore EMP3 expression. **B** Western blot showing EMP3 and EGFR levels in a panel of six patient-derived GSC lines. NCH1425 (EGFR-high) and NCH644 (EGFR-low) were further used in the study. **C, D** Chromosomal copy number plots of NCH1425 (C) and NCH644 (D) cells.

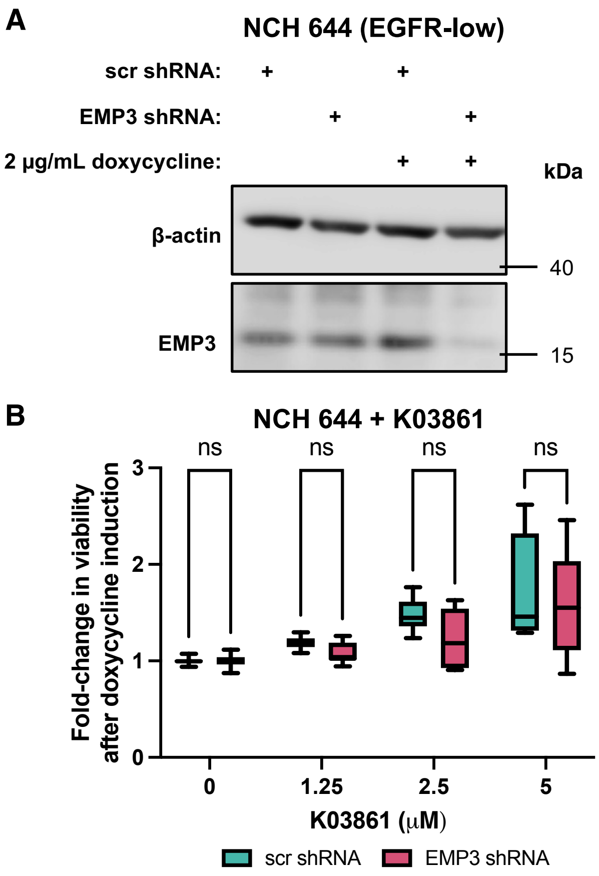

Figure S8. EMP3 silencing does not synergize with CDK2 inhibition in EGFR-low NCH644 GSCs.

A Western blots verifying successful EMP3 silencing in doxycycline-treated NCH644 GSCs transduced with inducible EMP3 shRNAs. **B** Fold-change in the viability of NCH644 GSCs after induction of shRNA expression and treatment with increasing concentrations of the CDK2 inhibitor K03861 (multiple Welch's t-test; ns – not significant).

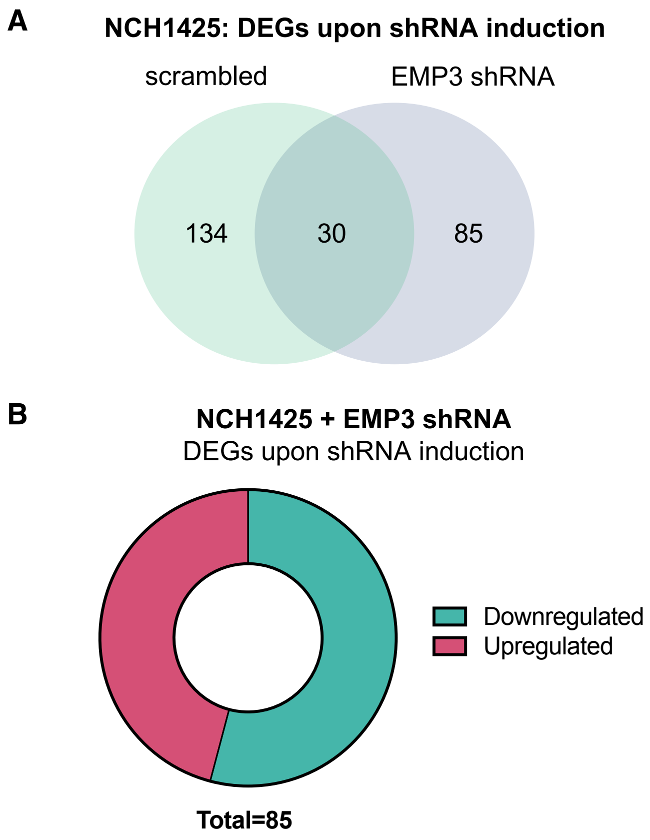

Figure S9. EMP3 silencing does not synergize with CDK2 inhibition in EGFR-low NCH644 GSCs.

A Venn diagram showing the DEGs induced upon expression of the scrambled or EMP3 shRNA in NCH1425 GSCs. **B** Donut chart showing the proportion of upregulated and downregulated genes among the 85 DEGs that are unique to EMP3-silenced NCH1425 cells.
